# Effects of collagen membranes enriched with in vitro-differentiated N1E-115 cells on rat sciatic nerve regeneration after end-to-end repair

**DOI:** 10.1186/1743-0003-7-7

**Published:** 2010-02-11

**Authors:** Sandra Amado, Jorge M Rodrigues, Ana L Luís, Paulo AS Armada-da-Silva, Márcia Vieira, Andrea Gartner, Maria J Simões, António P Veloso, Michele Fornaro, Stefania Raimondo, Artur SP Varejão, Stefano Geuna, Ana C Maurício

**Affiliations:** 1 Centro de Estudos de Ciência Animal (CECA), Instituto de Ciências e Tecnologias Agrárias e Agro-Alimentares (ICETA), Universidade do Porto (UP), Portugal; 2 Departamento de Clínicas Veterinárias, Instituto de Ciências Biomédicas Abel Salazar (ICBAS), Universidade do Porto (UP), Portugal; 3 Faculdade de Motricidade Humana (FMH), Universidade Técnica de Lisboa (UTL), Portugal; 4 Department of Clinical and Biological Sciences, University of Turin, Italy; 5 Departamento de Ciências Veterinárias, Universidade de Trás-os-Montes e Alto Douro (UTAD), Portugal

## Abstract

Peripheral nerves possess the capacity of self-regeneration after traumatic injury but the extent of regeneration is often poor and may benefit from exogenous factors that enhance growth. The use of cellular systems is a rational approach for delivering neurotrophic factors at the nerve lesion site, and in the present study we investigated the effects of enwrapping the site of end-to-end rat sciatic nerve repair with an equine type III collagen membrane enriched or not with N1E-115 pre-differentiated neural cells. After neurotmesis, the sciatic nerve was repaired by end-to-end suture (*End-to-End *group), end-to-end suture enwrapped with an equine collagen type III membrane (*End-to-EndMemb *group); and end-to-end suture enwrapped with an equine collagen type III membrane previously covered with neural cells pre-differentiated *in vitro *from N1E-115 cells (*End-to-EndMembCell *group). Along the postoperative, motor and sensory functional recovery was evaluated using extensor postural thrust (EPT), withdrawal reflex latency (WRL) and ankle kinematics. After 20 weeks animals were sacrificed and the repaired sciatic nerves were processed for histological and stereological analysis. Results showed that enwrapment of the rapair site with a collagen membrane, with or without neural cell enrichment, did not lead to any significant improvement in most of functional and stereological predictors of nerve regeneration that we have assessed, with the exception of EPT which recovered significantly better after neural cell enriched membrane employment. It can thus be concluded that this particular type of nerve tissue engineering approach has very limited effects on nerve regeneration after sciatic end-to-end nerve reconstruction in the rat.

## Background

Nerve regeneration is a complex biological phenomenon. In the peripheral nervous system, nerves can spontaneously regenerate without any treatment if nerve continuity is maintained (axonotmesis) whereas more severe type of injuries must be surgically treated by direct end-to-end surgical reconnection of the damaged nerve ends [1-3]. Unfortunately, the functional outcomes of nerve repair are in many cases unsatisfactory [4] thus calling for research in order to reveal more effective strategies for improving nerve regeneration. However, recent advances in neuroscience, cell culture, genetic techniques, and biomaterials provide optimism for new treatments for nerve injuries [5-17].

The use of materials of natural origin has several advantages in tissue engineering. Natural materials are more likely to be biocompatible than artificial materials. Also, they are less toxic and provide a good support to cell adhesion and migration due to the presence of a variety of surface molecules. Drawbacks of natural materials include potential difficulties in their isolation and controlled scale-up [11]. In addition to the use of intact natural tissues, a great deal of research has focused on the use of purified natural extracellular matrix (ECM) molecules, which can be modified to serve as appropriate scaffolding [11]. ECM molecules, such as laminin, fibronectin and collagen have also been shown to play a significant role in axonal development and regeneration [12,18-27]. For example, silicone tubes filled with laminin, fibronectin, and collagen led to a better regeneration over a 10 mm rat sciatic nerve gap compared to empty silicone controls [9]. Collagen filaments have also been used to guide regenerating axons across 20-30 mm defects in rats [23-27]. Further studies have shown that oriented fibers of collagen within gels, aligned using magnetic fields, provide an improved template for neurite extension compared to randomly oriented collagen fibers [28,29]. Finally, rates of regeneration comparable to those using a nerve autograft have been achieved using collagen tubes containing a porous collagen-glycosaminoglycan matrix [30-32]. Nerve regeneration requires a complex interplay between cells, ECM, and growth factors. The local presence of growth factors plays an important role in controlling survival, migration, proliferation, and differentiation of the various cell types involved in nerve regeneration [12-14,33]. Therefore, therapies with relevant growth factors received increasing attention in recent years although growth factor therapy is a difficult task because of the high biological activity (in pico- to nanomolar range), pleiotrophic effects (acting on a variety of targets), and short biological half-life (few minutes to hours) [34]. Thus, growth factors should be administered locally to achieve an adequate therapeutic effect with little adverse reactions and the short biological half-life of growth factors demands for a delivery system that slowly releases locally the molecules over a prolonged period of time. Employment of biodegradable membranes enriched with a cellular system producing neurotrophic factors has been suggested to be a rational approach for improving nerve regeneration after neurotmesis [11].

The aim of this study was thus to verify if rat sciatic nerve regeneration after end-to-end reconstruction can be improved by seeding *in vitro *differentiated N1E-115 neural cells on a type III equine collagen membrane and enwrap the membrane around the lesion site. The N1E-115 cell line has been established from a mouse neuroblastoma [35] and have already been used with conflicting results as a cellular system to locally produce and deliver neurotrophic factors [12-14,36,37]. *In vitro*, the N1E-115 cells undergo neuronal differentiation in response to dimethylsulfoxide (DMSO), adenosine 3', 5'-cyclic monophosphate (cAMP), or serum withdrawal [38-43,36,37,12-14]. Upon induction of differentiation, proliferation of N1E-115 cells ceases, extensive neurite outgrowth is observed and the membranes become highly excitable [38-43,36,37,12-14]. The interval period of 48 hours of differentiation was previously determined by measurement of the intracellular calcium concentration ([Ca^2+^] i). At this time point, the N1E-115 cells present already the morphological characteristics of neuronal cells but cell death due to increased [Ca^2+^] i is not yet occurring as described elsewhere [38-43,36,37,12-14].

## Methods

### Cell culture

The N1E-115 cells, clones of cells derived from the mouse neuroblastoma C-130035 retain numerous biochemical, physiological, and morphological properties of neuronal cells in culture [38-43,36,37,12-14]. N1E-115 neuronal cells were cultured in Petri dishes (around 2 × 10^6 ^cells) over collagen type III membranes (Gentafleece^®^, Resorba Wundversorgung GmbH + Co. KG, Baxter AG) at 37°C, 5% CO_2 _in a humidified atmosphere with 90% Dulbecco's Modified Eagle's Medium (DMEM; Gibco) supplemented with 10% fetal bovine serum (FBS, Gibco), 100 U/ml penicillin, and 100 μg/ml streptomycin (Gibco). The culture medium was changed every 48 hours and the Petri dishes were observed daily. The cells were passed or were supplied with differentiating medium containing 1.5% of DMSO once they reached approximately 80% confluence, mostly 48 hours after plating (and before the rats' surgery). The differentiating medium was composed of 96% DMEM supplemented with 2.5% of FBS, 100 U/ml penicillin, 100 μg/ml streptomycin and 1.5% of DMSO [12-14,36,37].

### Surgical procedure

Adult male Sasco Sprague Dawley rats (Charles River Laboratories, Barcelona, Spain) weighing 300-350 g, were randomly divided in 3 groups of 6 or 7 animals each. All animals were housed in a temperature and humidity controlled room with 12-12 hours light/dark cycles, two animals per cage (Makrolon type 4, Tecniplast, VA, Italy), and were allowed normal cage activities under standard laboratory conditions. The animals were fed with standard chow and water *ad libitum*. Adequate measures were taken to minimize pain and discomfort taking in account human endpoints for animal suffering and distress. Animals were housed for two weeks before entering the experiment. For surgery, rats were placed prone under sterile conditions and the skin from the clipped lateral right thigh scrubbed in a routine fashion with antiseptic solution. The surgeries were performed under an M-650 operating microscope (Leica Microsystems, Wetzlar, Germany). Under deep anaesthesia (ketamine 90 mg/Kg; xylazine 12.5 mg/Kg, atropine 0.25 mg/Kg i.m.), the right sciatic nerve was exposed through a skin incision extending from the greater trochanter to the mid-thigh distally followed by a muscle splitting incision. After nerve mobilisation, a transection injury was performed (neurotmesis) immediately above the terminal nerve ramification using straight microsurgical scissors. Rats were then randomly assigned to three experimental groups. In one group (*End-to-End*), immediate cooptation with 7/0 monofilament nylon epineurial sutures of the 2 transected nerve endings was performed, in a second group (*End-to-EndMemb*) nerve transection was reconstructed by end-to-end suture, like in the first group, and then enveloped by a membrane of equine collagen type III. In a third group (*End-to-EndMembCell*) animals received the same treatment as the previous group but with equine collagen type III membranes covered with neural cells differentiated *in vitro*. Sciatic nerves from the contralateral site were left intact in all groups and served as controls. To prevent autotomy, a deterrent substance was applied to rats' right foot [44,45]. The animals were intensively examined for signs of autotomy and contracture during the postoperative and none presented severe wounds, infections or contractures. All procedures were performed with the approval of the Veterinary Authorities of Portugal in accordance with the European Communities Council Directive of November 1986 (86/609/EEC).

### Evaluation of motor performance (EPT) and nociceptive function (WRL)

Motor performance and nociceptive function were evaluated by measuring extensor postural thrust (EPT) and withdrawal reflex latency (WRL), respectively. The animals were tested pre-operatively (week-0), at weeks 1, 2, and every two weeks thereafter until week-20. The animals were gently handled, and tested in a quiet environment to minimize stress levels. The EPT was originally proposed by Thalhammer and collaborators, in 1995 [46] as a part of the neurological recovery evaluation in the rat after sciatic nerve injury. For this test, the entire body of the rat, excepting the hind-limbs, was wrapped in a surgical towel. Supporting the animal by the thorax and lowering the affected hind-limb towards the platform of a digital balance, elicits the EPT. As the animal is lowered to the platform, it extends the hind-limb, anticipating the contact made by the distal metatarsus and digits. The force in grams (g) applied to the digital platform balance (model TM 560; Gibertini, Milan, Italy) was recorded. The same procedure was applied to the contralateral, unaffected limb. Each EPT test was repeated 3 times and the average result was considered. The normal (unaffected limb) EPT (NEPT) and experimental EPT (EEPT) values were incorporated into an equation (Equation 1) to derive the functional deficit (varying between 0 and 1), as described by Koka and Hadlock, in 2001 [47].(1)

To assess the nociceptive withdrawal reflex (WRL), the hotplate test was modified as described by Masters and collaborators [48]. The rat was wrapped in a surgical towel above its waist and then positioned to stand with the affected hind paw on a hot plate at 56°C (model 35-D, IITC Life Science Instruments, Woodland Hill, CA). WRL is defined as the time elapsed from the onset of hotplate contact to withdrawal of the hind paw and measured with a stopwatch. Normal rats withdraw their paws from the hotplate within 4.3 s or less [49]. The affected limbs were tested 3 times, with an interval of 2 min between consecutive tests to prevent sensitization, and the three latencies were averaged to obtain a final result [50,51]. If there was no paw withdrawal after 12 s, the heat stimulus was removed to prevent tissue damage, and the animal was assigned the maximal WRL of 12 s [52].

### Kinematic Analysis

Ankle kinematics during the stance phase of the rat walk was recorded prior nerve injury (week-0), at week-2 and every 4 weeks during the 20-week follow-up time. Animals walked on a Perspex track with length, width and height of respectively 120, 12, and 15 cm. In order to ensure locomotion in a straight direction, the width of the apparatus was adjusted to the size of the rats during the experiments, and a darkened cage was placed at the end of the corridor to attract the animals. The rats gait was video recorded at a rate of 100 images per second (JVC GR-DVL9800, New Jersey, USA). The camera was positioned perpendicular to the mid-point of the corridor length at a 1-m distance thus obtaining a visualization field of 14-cm wide. Only walking trials with stance phases lasting between 150 and 400 ms were considered for analysis, since this corresponds to the normal walking velocity of the rat (20-60 cm/s) [52-54]. The video images were stored in a computer hard disk for latter analysis using an appropriate software APAS^® ^(Ariel Performance Analysis System, Ariel Dynamics, San Diego, USA). 2-D biomechanical analysis (sagittal plan) was carried out applying a two-segment model of the ankle joint, adopted from the model firstly developed by Varejão and collaborators [52-55]. Skin landmarks were tattooed at points in the proximal edge of the tibia, in the lateral malleolus and, in the fifth metatarsal head. The rats' ankle angle was determined using the scalar product between a vector representing the foot and a vector representing the lower leg. With this model, positive and negative values of position of the ankle joint indicate dorsiflexion and plantarflexion, respectively. For each stance phase the following time points were identified: initial contact (IC), opposite toe-off (OT), heel-rise (HR) and toe-off (TO) [52-55], and were time normalized for 100% of the stance phase. The normalized temporal parameters were averaged over all recorded trials. Angular velocity of the ankle joint was also determined where negative values correspond to dorsiflexion. Four steps were analysed for each animal [55].

### Histological and Stereological analysis

A 10-mm-long segment of the sciatic nerve distal to the site of lesion was removed, fixed, and prepared for quantitative morphometry of myelinated nerve fibers. A 10-mm segment of uninjured sciatic nerve was also withdrawn from control animals (N = 6). The harvested nerve segments were immersed immediately in a fixation solution containing 2.5% purified glutaraldehyde and 0.5% saccarose in 0.1 M Sorensen phosphate buffer for 6-8 hours. Specimens were processed for resin embedding as described in details elsewhere [56,57]. Series of 2-μm thick semi-thin transverse sections were cut using a Leica Ultracut UCT ultramicrotome (Leica Microsystems, Wetzlar, Germany) and stained by Toluidine blue for stereological analysis of regenerated nerve fibers. The slides were observed with a DM4000B microscope equipped with a DFC320 digital camera and an IM50 image manager system (Leica Microsystems, Wetzlar, Germany). One semi-thin section from each nerve was randomly selected and used for the morpho-quantitative analysis. The total cross-sectional area of the nerve was measured and sampling fields were then randomly selected using a protocol previously described [57-59]. Bias arising from the "edge effect" was coped with the employment of a two-dimensional disector procedure which is based on sampling the "tops" of fibers [60,61]. Mean fiber density in each disector was then calculated by dividing the number of nerve fibers counted by the disector's area (N/mm^2^). Finally, total fiber number (N) in the nerve was estimated by multiplying the mean fiber density by the total cross-sectional area of the whole nerve. Two-dimensional disector probes were also used for the unbiased selection of a representative sample of myelinated nerve fibers for estimating circle-fitting diameter and myelin thickness. Precision and accuracy of the estimates were evaluated by calculating the coefficient of variation (CV = SD/mean) and coefficient of error (CE = SEM/mean) [57-59].

### Statistical analysis

Two-way mixed factorial ANOVA was used to test for the effect of time in the *End-to-End *group (within subjects effect; 12 time points) and experimental groups (between subjects effect, 3 groups). The sphericity assumption was evaluated by the Mauchly's test and when this test could not be computed or when sphericity assumption was violated, adjustment of the degrees of freedom was done with the Greenhouse-Geiser's epsilon. When time main effect was significant (within subjects factor), simple planned contrasts (General Linear Model, simple contrasts) were used to compare pooled data across the three experimental groups along the recovery with data at week-0. When a significant main effect of treatment existed (between subjects factor), pairwise comparisions were carried out using the Tukey's HSD test. At week-0, kinematic data was recorded only from the *End-to-End *group so the main effect of time was evaluated only in this group. Evaluation of the main effect of treatment on ankle motion variables used only data after nerve injury. In this case, and when appropriate, pairwise comparisons were made using the Tukey's HSD test. Statistical comparisons of stereological morpho-quantitative data on nerve fibers were accomplished with one-way ANOVA test. Statistical significance was established as p < 0.05. All statistical procedures were performed by using the statistical package SPSS (version 14.0, SPSS, Inc) except stereological data that were analysed using the software "*Statistica per discipline bio-mediche*" (McGraw-Hill, Milan, Italy). All data in this study is presented as mean ± standard error of the mean (SEM).

## Results

### Motor deficit and Nociception function

#### Motor deficit (EPT)

**Figure 1:**
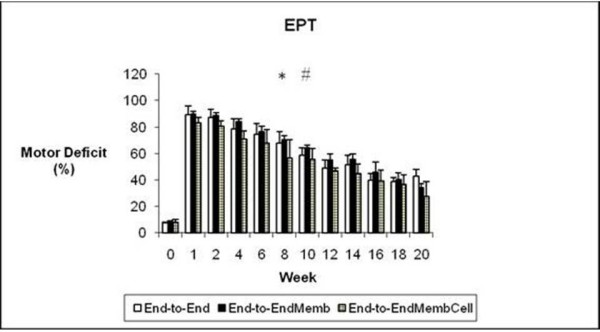
**Weekly values of the percentage of motor deficit obtained by the Extensor Postural Thrust (EPT) test**. * Significantly different from week-0 all groups pooled together (p < 0.05). # Group *End-to-EndMembCell *significantly different from the other groups (p < 0.05). Results are presented as mean and standard error of the mean (SEM).

Before sciatic injury, EPT was similar in both hindlimbs in all experimental groups (figure 1). In the first week after sciatic nerve transection, near total EPT loss was observed in the operated hindlimb, leading to a motor deficit ranging between 83 to 90%. The EPT response steadily improved during recovery but at week-20 the EPT values of the injured side were still significantly lower compared to values at week-0 (p < 0.05). A significant main effect for treatment was found [F_(2,17) _= 14.202; p = 0.000], with pairwise comparisons showing significantly better recovery of the EPT response in the *End-to-EndMembCell *group when compared to the other two experimental groups (p < 0.05). At week-20, motor deficit decreased to 27% in the *End-to-EndMembCell *and to 34% and 42% in the *End-to-End *and *End-to-EndMemb *groups, respectively (figure 1).

#### Nociception function (WRL)

**Figure 2:**
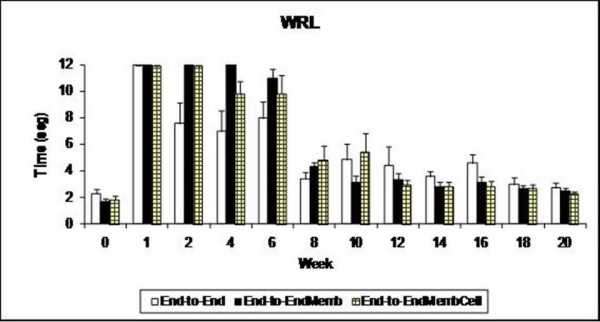
**Weekly values of the withdrawal reflex latency test**. At week-1 all animals failed in responding to the noxious thermal stimulus within the 12 sec cut-off time. No differences between the percentages of motor deficit obtained by the Extensor Postural Thrust (EPT) test. * Significantly different from week-0 all groups pooled together (p < 0.05). Results are presented as mean and standard error of the mean (SEM).

In the week after sciatic transaction, all the animals presented a severe loss of sensory and nociception function acutely after sciatic nerve transection and the WRL test has to be interrupted at the 12 s-cutoff time (figure 2). During the following weeks there was recovery in paw nociception which was more clearly seen between weeks 6 and 8 post-surgery. At week-6, half of the animals still had no withdrawal response to the noxious thermal stimulus in the operated side, which is in contrast with week-8, when all animals presented a consistent, although delayed, response. Despite such improvement in WRL response, contrast analysis showed persistence of sensory deficit in all groups by the end of the 20-weeks recovery time (p < 0.05). No differences between the groups was observed in the level of WRL impairment after the sciatic nerve transection [F_(2,17) _= 1.563; p = 0.238].

### Kinematics Analysis

**Figure 3:**
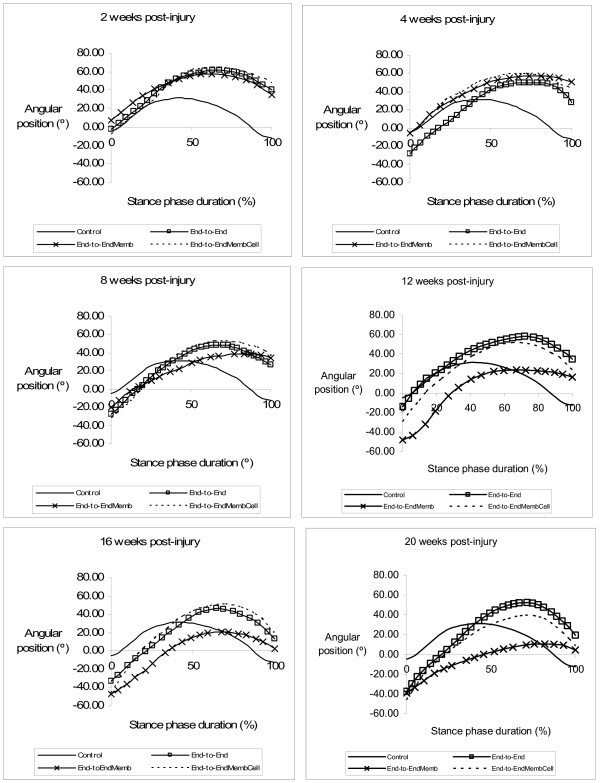
**Kinematics plots in the sagittal plane for the angular position (°) of the ankle as it moves through the stance phase, during the healing period of 20 weeks**. The mean of each group is plotted.

**Figure 4:**
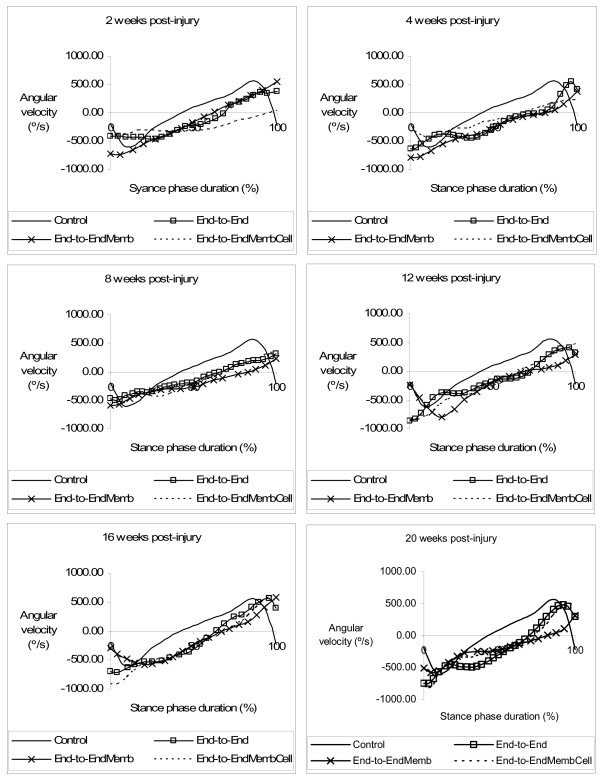
**Kinematics plots in the sagittal plane for the angular velocity (°/s) of the ankle as it moves through the stance phase, during the healing period of 20 weeks**. The mean of each group is plotted.

Figures 3 and 4 display the mean plots, respectively for ankle joint angle and ankle joint velocity during the stance phase of the rat walk. Comparisons to the normal ankle motion can only be draw for the *End-to-End *group for reasons explained in the Methods section. In the weeks following sciatic nerve transection, ankle joint motion became severely abnormal, particularly throughout the second half of stance corresponding to the push-off sub-phase. In clear contrast to the normal pattern of ankle movement, at week-2 post-injury animals were unable to extend this joint and dorsiflexion continued increasing during the entire stance, which is explained by the paralysis of plantarflexor muscles. The pattern of the ankle joint motion seemed to have improved only slightly during recovery. Contrast analysis was performed for each of the kinematic parameters (tables 1 and 2) with somewhat different results. For OT velocity and HR angle no differences existed before and after sciatic nerve transection, whereas for OT angle differences from pre-injury values were significant only at weeks 2 and 16 of recovery (p < 0.05). The angle at IC showed a unique pattern of changes, being unaffected at week-2 post-injury and altered from normal in the following weeks of recovery. Probably the most consistent results are those of HR velocity, TO angle and TO velocity. These parameters were affected immediately after the nerve injury and remained abnormal along the entire 20-weeks recovery period (p < 0.05). The effect of the different tissue engineering strategies was assessed comparing the kinematic data of the experimental groups only during the recovery period (see Methods). Statistical analysis demonstrated that the collagen membrane and the cells had no or little effect on ankle motion pattern recovery. Generally, no differences in the kinematic parameters were found between the groups. Exceptions were IC velocity in the *End-to-EndMembCell *group, which was different from the other two groups (p < 0.05), and OT angle in the *End-to-EndMemb *group that was also different from the other two groups (p < 0.05).

**Table 1:** Ankle kinematics and stance duration analysis were carried out prior to nerve injury (week-0), at week-2, and every 4 weeks during the 20-week follow-up period.

Temporal Parameter		Week 0	Week 2	Week 4	Week 8	Week 12	Week 16	Week 20
**IC**	End-to-End	-4.84 ± 3.00	2.70 ± 1.29	-30.11 ± 5.38	-20.88 ± 4.22	-28.36 ± 3.84	-38.92 ± 4.82	-52.83 ± 6.46
	End-to-EndMemb		7.19 ± 2.94	-6.25 ± -2.55	-19.65 ± -8.02	-48.15 ± -19.66	-46.87 ± -19.13	-39.03 ± -15.93
	End-to-EndMembCell		4.95 ± 0.68	-31.59 ± 12.98	-23.79 ± 2.47	-29.57 ± 5.74	-42.25 ± 11.38	-46.04 ± 9.29

**OT**	End-to-End	25.65 ± 1.08	36.74 ± 4.71	18.75 ± 2.81	25.58 ± 8.88	24.79 ± 2.62	4.71 ± 4.35	16.61 ± 3.96
	End-to-EndMemb		20.37 ± 4.61	21.54 ± 9.92	9.78 ± 18.75	4.26 ± 18.44	-19.51 ± 16.74	-18.70 ± 20.74
	End-to-EndMembCell		33.15 ± 2.98	25.08 ± 4.86	28.15 ± 8.57	24.54 ± 8.91	21.75 ± 5.76	16.47 ± 6.73

**HR**	End-to-End	30.67 ± 2.44	51.20 ± 4.50	40.03 ± 2.21	36.82 ± 5.96	34.01 ± 5.87	34.30 ± 3.35	40.37 ± 4.75
	End-to-EndMemb		52.46 ± 2.16	48.04 ± 5.49	29.65 ± 22.49	20.89 ± 21.10	11.57 ± 20.18	4.16 ± 26.71
	End-to-EndMembCell		54.80 ± 2.44	44.16 ± 3.90	46.41 ± 6.68	43.69 ± 6.09	41.53 ± 6.66	29.08 ± 7.55

**TO**	End-to-End	-12.27 ± 7.01	41.49 ± 3.23	39.33 ± 3.03	27.28 ± 1.38	28.04 ± 3.11	15.02 ± 3.78	12.16 ± 3.43
	End-to-EndMemb		35.56 ± 1.69	50.77 ± 4.41	34.64 ± 21.86	16.36 ± 20.87	2.12 ± 18.58	4.60 ± 25.45
	End-to-EndMembCell		48.10 ± 1.60	44.76 ± 4.17	36.40 ± 6.11	23.37 ± 4.02	18.64 ± 5.76	8.71 ± 4.20

Values of the ankle angular position (°) at initial contact (IC); opposite toe-off (OT); heel-rise (HR); toe-off (TO) of the stance phase. Results are presented as mean and standard error of the mean (SEM). N corresponds to the number of rats within the experimental group.

**Table 2:** Ankle kinematics and stance duration analysis were carried out prior to nerve injury (wek-0), at week-2, and every 4 weeks during the 20-week follow-up period.

Temporal Parameter		Week 0	Week 2	Week 4	Week 8	Week 12	Week 16	Week 20
**IC**	End-to-End	-194.15 ± 44.35	-448.33 ± 66.25	-604.86 ± 66.95	-351.64 ± 73.81	-639.43 ± 120.70	-809.90 ± 88.67	-647.63 ± 81.94
	End-to-EndMemb		-728.48 ± -297.40	-785.62 ± -320.73	-593.43 ± -242.26	-234.56 ± -95.76	-302.06 ± -123.31	-514.54 ± -210.06
	End-to-EndMembCell		-557.10 ± 224.85	-505.97 ± 108.58	-845.50 ± 160.47	-933.63 ± 57.41	-914.05 ± 80.10	-903.24 ± 74.55

**OT**	End-to-End	-270.35 ± 19.65	-273.97 ± 47.92	-385.24 ± 37.99	-399.28 ± 34.85	-530.42 ± 68.29	-460.90 ± 66.08	-414.22 ± 35.18
	End-to-EndMemb		-641.95 ± -262.08	-528.60 ± -215.80	-321.92 ± -131.42	-449.49 ± -183.50	-582.66 ± -237.87	-411.32 ± -167.92
	End-to-EndMembCell		-357.80 ± 43.21	-495.68 ± 82.13	-372.17 ± 33.65	-467.31 ± 76.14	-471.29 ± 20.94	-278.67 ± 20.71

**HR**	End-to-End	53.25 ± 40.58	-177.64 ± 41.45	-246.61 ± 11.49	-333.55 ± 16.41	-280.20 ± 24.32	-322.47 ± 18.46	-322.86 ± 23.80
	End-to-EndMemb		-265.19 ± 28.15	-301.10 ± 65.99	-268.48 ± 64.92	-114.06 ± 82.52	-327.76 ± 86.17	-222.91 ± 51.33
	End-to-EndMembCell		-353.02 ± 24.39	-285.60 ± 21.13	-190.41 ± 9.69	-286.61 ± 57.54	-283.54 ± 12.24	-216.29 ± 21.36

**TO**	End-to-End	-221.38 ± 91.28	322.87 ± 109.64	327.44 ± 31.23	399.79 ± 82.70	403.59 ± 57.88	444.05 ± 78.95	193.03 ± 130.15
	End-to-EndMemb		554.31 ± 69.27	384.52 ± 66.65	227.17 ± 123.13	281.98 ± 79.91	577.14 ± 155.51	311.14 ± 197.88
	End-to-EndMembCell		248.78 ± 30.40	420.52 ± 28.58	355.25 ± 43.90	466.17 ± 43.54	551.88 ± 43.74	460.01 ± 51.01

Values of the ankle angular velocity (°/sec) at initial contact (IC); opposite toe-off (OT); heel-rise (HR); toe-off (TO) of the stance phase. Results are presented as mean and standard error of the mean (SEM). N corresponds to the number of rats within the experimental group.

### Histological and Stereological Analysis

**Figure 5:**
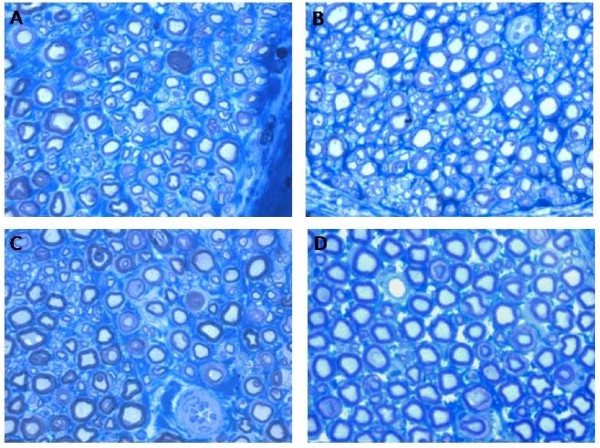
**Representative high resolution photomicrographs of nerve fibers form regenerated (A-C) and normal (D) rat sciatic nerves**. A: *End-to-End*. B:*End-to-EndMemb*. C:*End-to-EndMembCell*. Magnification = × 1,500.

Figure 5 shows representative light micrographs of the regenerated sciatic nerves of the three groups (figure 5A-C) and control sciatic normal nerves (figure 5D). As expected, regeneration of axons was organized in many smaller fascicles in comparison to controls. The results of the stereological analysis of myelinated nerve fibers are reported in Table 3. Statistical analysis by ANOVA test revealed no significant (p > 0.05) difference regarding any of the morphological parameters investigated in the regenerated axons from the three experimental groups. On the other hand, comparison between regenerated and control nerves showed, as expected, the presence of a significantly (p < 0.05) higher density and total number of myelinated axons in experimental groups accompanied by a significantly (p < 0.05) lower fiber diameter.

**Table 3:** Stereological quantitative assessment density, total number, diameter and myelin thickness of regenerated sciatic nerve fibers at week-20 after neurotmesis.

	N/mm^2^ Density	N Number	D Fiber diameter (μm)
*EndtoEnd*	20,612 ± 1,607	14,624 ± 1,642	4.06 ± 0.30
*EndtoEndMemb*	23,575 ± 1,018	15,101 ± 1,172	3.87 ± 0.18
*EndtoEndMembCell*	22,394 ± 1,750	14,467 ± 1,524	3.96 ± 0.21
*Control*	15,905 ± 287	7,666 ± 190	6.66 ± 0.12

Values are presented as mean ± SEM.

## Discussion

Transected peripheral nerves can regenerate provided that a connection is available between the proximal and distal severed stumps and, when no substance loss occurred, surgical treatment consists in direct end-to-end suturing of the nerve ends [1-3,62,63]. However, in spite of the progress of microsurgical nerve repair, the outcome of nerve reconstruction is still far from being optimal^4^. Since during regeneration axons require neurotrophic support, they could benefit from the presence of a growth factors delivery cell system capable of responding to stimuli of the local environment during axonal regeneration.

In the present study, we aimed at investigating the effects of enwrapping the site of end-to-end rat sciatic nerve repair with equine type III collagen nerve membranes either alone or enriched with N1E-115 pre-differentiated into neural cells in the presence of 1.5% of DMSO. The rationale for the utilization of the N1E-115 cells was to take advantages of the properties of these cells as a neural-like cellular source of neurotrophic factors [12-14,36,37].

Results showed that enwrapment with a collagen membrane, with or without neural cell enrichment, did not lead to any significant improvement in most of functional and stereological predictors of nerve regeneration that we have assessed. The only exception was represented by motor deficit recovery which was significantly improved after lesion site enwrapment with membrane enriched with neural cells pre-differentiated from N1E-115 cell line.

Natural tissues possess several advantages when compared to synthetic materials, when use to reconstruct peripheral nerves after injury. Natural materials are more likely to be biocompatible than artificial materials, are less toxic, and provide a support structure to promote cell adhesion and migration. Drawbacks, on the other hand, include potential difficulties with isolation and controlled scale-up. In addition to intact acellular tissues, a great deal of research has focused on the use of purified natural ECM proteins and glycosaminoglycans, which can be modified to serve as appropriate scaffolding. ECM molecules, such as laminin, collagen, and fibronectin, have been shown to play a significant role in axonal development and repair in the body [19,24]. There are a number of examples in which the ECM proteins laminin, fibronectin, and collagen have been used for nerve repair applications [12,18-27]. For example, silicone tubes filled with laminin, fibronectin, and collagen show improved regeneration over a 10 mm rat sciatic nerve gap compared to empty silicone controls [9]. Collagen filaments have also been used to guide regenerating axons across 20-30 mm defects in rats [23,26,27]. Further studies have shown that oriented fibers of collagen within gels, aligned using magnetic fields, provide an improved template for neurite extension compared to randomly oriented collagen fibers [28,29]. Rates of regeneration after neurotmesis comparable to those using a nerve autograft have been achieved using collagen tubes containing a porous collagen-glycosaminoglycan matrix [31,32].

Results of this study contribute to the lively debate about the employment of cell transplantation for improving post-traumatic nerve regeneration [64,65]. Actually, a great enthusiasm among researchers and especially the public opinion has risen over the last years about cell-based therapies in Regenerative Medicine [66-68] and there seems to be widespread conviction that this type of therapy is not only effective but also very safe in comparison to other pharmacological or surgical therapeutic approaches. By contrast, recent studies showed that cell-based therapy might be ineffective for improving nerve regeneration [66-69], and results of the present study are in line with these observations. Recently, it has even been shown that N1E-115 cell transplantation can also have negative results by hindering the nerve regeneration process after tubulisation repair [12]. Of course, the choice of the cell type to be used for transplantation is very important for the therapeutic success and use of another cell type could have led to better results, especially when the cellular system of choice is derived from autologous or heterologous stem cells1 [1,12,15-17,64,70]. Moreover, the construction of more appropriate tube-guides with integrated growth factor delivery systems and/or cellular components could improve the effectiveness of nerve tissue engineering. In fact, single-molded tube guides may not give sufficient control over both the mechanical properties and the delivery of bioactive agents. More complex devices will be needed, such as multilayered tube guides where growth factors are entrapped in polymer layers with varying physicochemical properties or tissue engineered tube guides containing viable stem cells [1,12,15-17,64,70]. The combination of two or more growth factors will likely exert a synergistic effect on nerve regeneration, especially when the growth factors belong to different families and act via different mechanisms. Combinations of growth factors can be expected to enhance further nerve regeneration, particularly when each of them is delivered at individually tailored kinetics [11,12,15-17,64,70,71]. The determination and control of suitable delivery kinetics for each of several growth factors will constitute a major hurdle both technically and biologically with the biological hurdle lying in the compliance with the naturally occurring cross talk between growth factors and cells. A solution to this problem may be the use of autologous stem cells because they can synthesize several growth factors and differentiate into Schwann cells which are critical for very long gaps [11,12,15-17,64,70,71].

Previous work already published by other research groups, point out a very interesting source of stem cells for nerve regeneration of peripheral nerve and spinal cord. They developed hair follicle pluripotent stem cells (hfPS) and have shown that these cells can differentiate to neurons, glial cells *in vitro*, and other cell types, and can promote nerve and spinal cord regeneration *in vivo*. These cells are located above the hair follicle bulge (hfPS cell area) and are nestin and CD34 positive, and keratin 15 negative [72-75]. The mouse hfPS cells were implanted into the gap region of the severed sciatic and tibial nerve of mice. These cells, after 6-8 weeks, transdifferentiated largely into Schwann cells. Also, blood vessels formed a network around the joined sciatic and tibial nerve. Function of the rejoined sciatic and tibial nerve was confirmed by contraction of the gastrocnemius muscle upon electrical stimulation and by walking track analysis [73-75]. hfPS cells can promote axonal growth and functional recovery after peripheral nerve injury, offering an important opportunity for future clinical application. These hfPS cells, in contrast to Embrionic stem cells, N1E-115 cells after *in vitro *differentiation and induced pluripotent stem cells, do not require any genetic manipulation, are readily accessible from any patient, and lack the ethical issues, do not form tumors.

## Competing interests

The authors declare that they have no competing interests.

## Authors' contributions

SA, APV and ASPV carried out the kinematic collecting data and the kinematic data analysis, participated in the functional data analysis, JMR and ALL carried out the animal surgeries, euthanasia, preparation of samples for histological and stereological analysis and participated in the functional evaluation analysis, PASADS carried out all the statistical analysis, the interpretation of kinematic data and participated in the paper draft, MV, AGand MJS performed the functional evaluation and analysis and were responsible for keeping the experimental animals, MF, SR, and SG performed the histological and stereological analysis, ACM carried out the animal surgeries euthanasia and preparation of samples for histological and stereological analysis. ACM together with SG and PASADS designed and coordinated the study, elaborated the manuscript and were responsible for the funding acquisition. All the authors read and approved the final manuscript.
